# Bacteria-ball in the urinary tract: a rare entity

**DOI:** 10.1259/bjrcr.20200039

**Published:** 2020-05-21

**Authors:** Lim Tze Ying Benjamin, Wai Loon Yam, Angeline Choo Choo Poh, Victor Ng, Sey Kiat Lim, Kok Kit Ng

**Affiliations:** 1 Department of Urology, Changi General Hospital, Singapore; 2 Department of Diagnostic Radiology, Changi General, Hospital, Singapore; 3 Department of Labaratory Medicine, Changi General, Hospital, Singapore

## Abstract

A bacterial mass in the urinary tract is a very rare entity. We report the first case of a bacterial ball within the urinary tract of a patient with diabetic cystopathy on long term urinary indwelling catheter. She presented with fever and gross haematuria. CT scan of abdomen and pelvis revealed a gas containing hyperdense mass within the bladder suspicious of bladder stone. The lesion was resected, and histopathology revealed a matrix of acellular materials with bacteria colony.

## Case

An 82-year-old Chinese Female presented with fever, gross haematuria, and lower abdominal pain for 1 day. Her past medical history includes diabetes mellitus, hypertension, and chronic kidney disease. Functionally, she is non-ambulant, and bed bound due to previous right hip fracture. She is under follow up with urology for diabetic cystopathy and 6 weekly change of long-term indwelling urinary catheter. Her urinary catheter was changed 1 month prior to admission. She has no previous surgical intervention to the urinary tract.

## Initial evaluation

Physical examination revealed lower suprapubic tenderness. Renal punch was negative on both sides. Blood test results showed: haemoglobin 9.7 g/dL, white blood cell count 12.8 × 10^3^/µL. Her glycaemic control is poor as evidenced by HBA1c of 10.3%, fasting blood glucose levels of 11.0 mmol/L and post-prandial blood glucose levels of 21.0 mmol/L. Her calcium, phosphate and uric acid levels were within normal limits. She has mild acute kidney injury with a serum creatinine was elevated at 148 μmol/L. Urinalysis showed haemopyuria, positive leukocyte esterase, and negative for nitrite test. Urine culture revealed mixed bacterial growth of more than three different species including *Enterococcus faecalis*. Blood culture had no bacterial growth. Urine cytology revealed no malignant cells. A plain KUB X-ray performed showed an opacity in the left central side of the pelvis consistent with the ‘’popcorn’’ calcification of an involuted fibroid. The colon and rectum were markedly faecal loaded. She was empirically treated with intravenous Ceftriaxone. A non-contrasted CT abdomen and pelvis revealed a large hyperdense mass with pockets of gas measuring 3.8 × 3.7 x 1.7 cm (Hounsfield unit of 350–422). Foleys catheter was seen *in situ*. Bladder wall was diffusely thickened. There is a small 6 mm calculus in the right kidney. The upper urinary tract was otherwise unremarkable.

**Figure 1.**
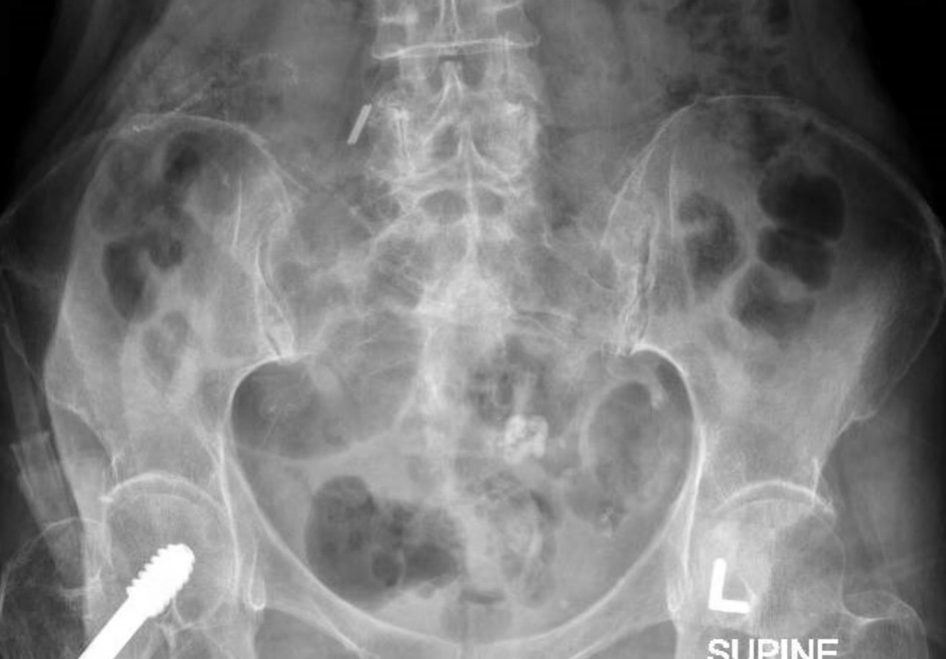
Initial KUB radiograph revealed no obvious urinary tract calculus. Opacity in the left central side of the pelvis consistent with ‘’popcorn’’ calcification of an involuted fibroid.

**Figure 2.**
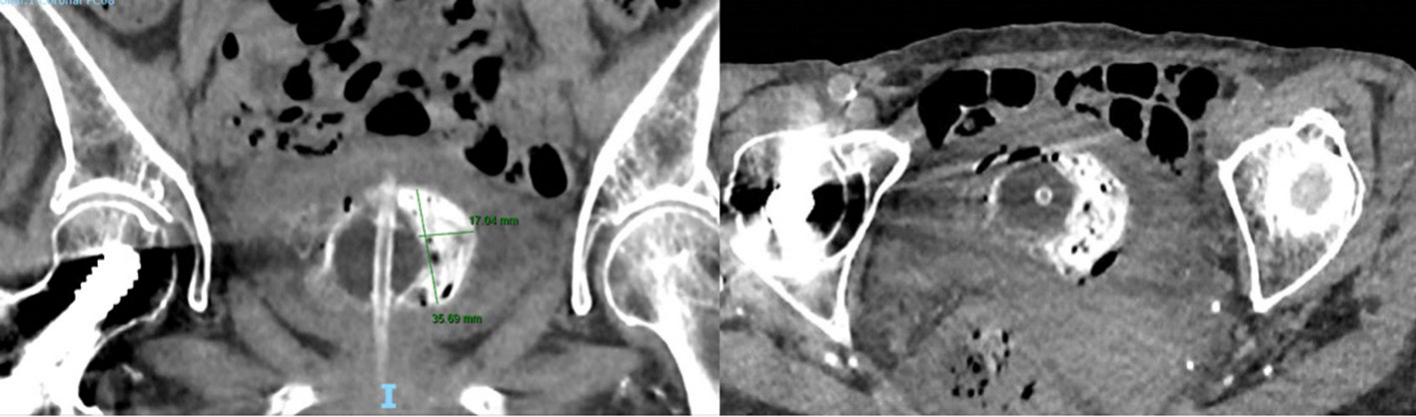
A hyperdense mass with pockets of gas in the bladder adjacent to the balloon of the Foleys catheter on coronal (a) and axial (b) pelvic CT images.

## Management

Manual bladder washout performed under ultrasound guidance with a size 24 Fr catheter evacuated 100 cc of clots. She was placed on continuous bladder irrigation. She responded well to antibiotics and was afebrile after 2 days of antibiotics. Her inflammatory markers were down trending and serum creatinine return to baseline. The bladder irrigation was stopped, and her urine remained clear. She was discharged back to nursing home and planned for an elective cystolithotripsy. However, she was re-admitted again a week later recurrent gross haematuria. Manual bladder washout was done and she was placed on continuous bladder irrigation. Repeat urine culture was negative. She was planned for cystolithotripsy in the same admission. However, intraoperative rigid cystoscopy revealed a soft cream coloured material densely adherent to the bladder mucosa. Bladder mucosa was severely trabeculated with multiple small diverticula. Initial attempts to aspirate and washout the mass with Toomey syringe was unsuccessful. A 26 Fr continuous flow resectoscope was used to dislodge the bacterial ball from the mucosa. It is then resected to smaller pieces with Bipolar energy and washed out. On resection, we noted certain areas of the bacterial ball exhibit laminated appearance. A random biopsy of posterior bladder wall was taken at the end of the procedure. Her urine was subsequently clear and post-operative stay was uneventful. Intraoperative tissue sent for aerobic culture revealed *Enterococcus faecalis* and mixed Gram-negative bacilli enteric organisms. Histology of tissue sent showed bacteria, small amounts of necrotic debris and some fibrinous exudates. No hyphae or fungal elements were seen. Bladder biopsy showed acute on chronic inflammatory picture. She was discharged back to nursing home with a silicone indwelling urinary catheter.

**Figure 3.**
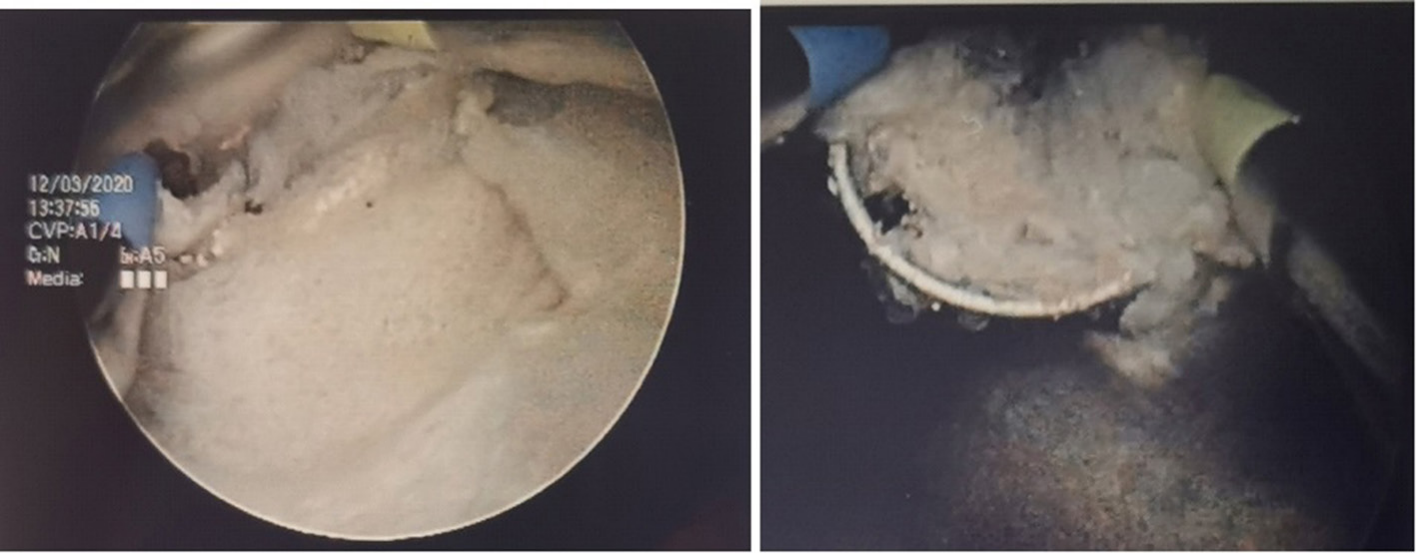
Cream coloured mass within the bladder cavity. Bipolar resectoscope is used to resect the lesion to smaller fragments.

**Figure 4.**
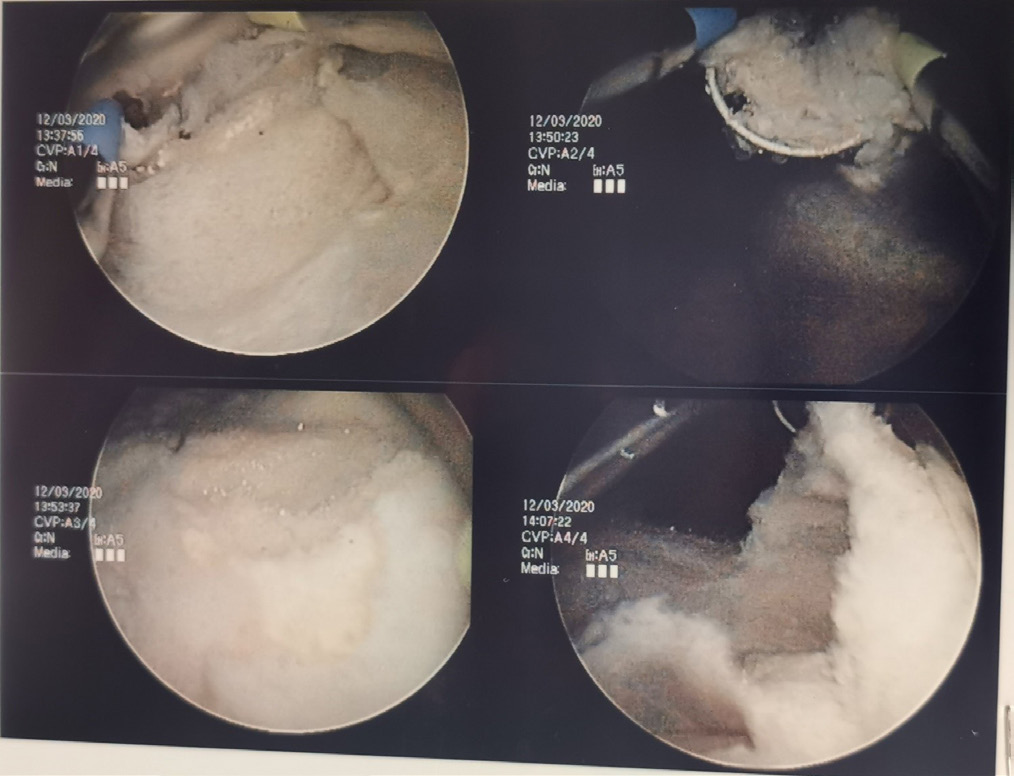
Certain areas of the bacterial ball exhibit laminated appearance, giving rise to gas seen on CT scan.

**Figure 5.**
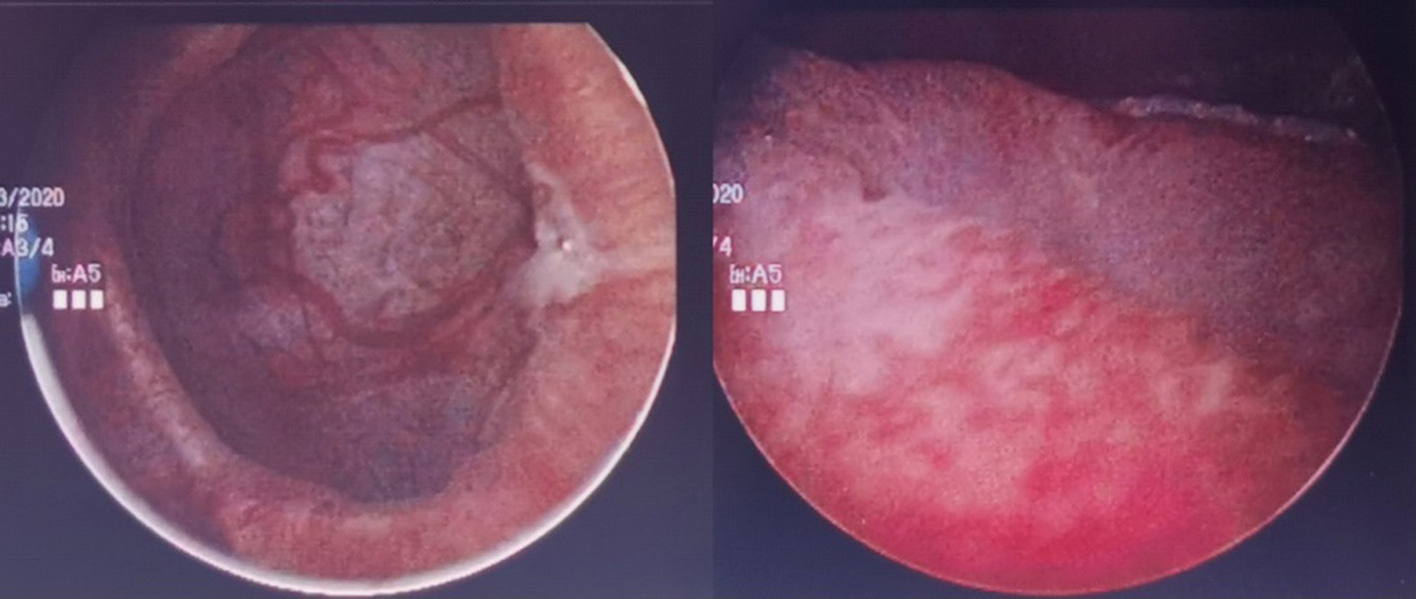
Cystoscopy findings showed a severely trabeculated bladder mucosa with multiple diverticula and cystitis changes.

**Figure 6.**
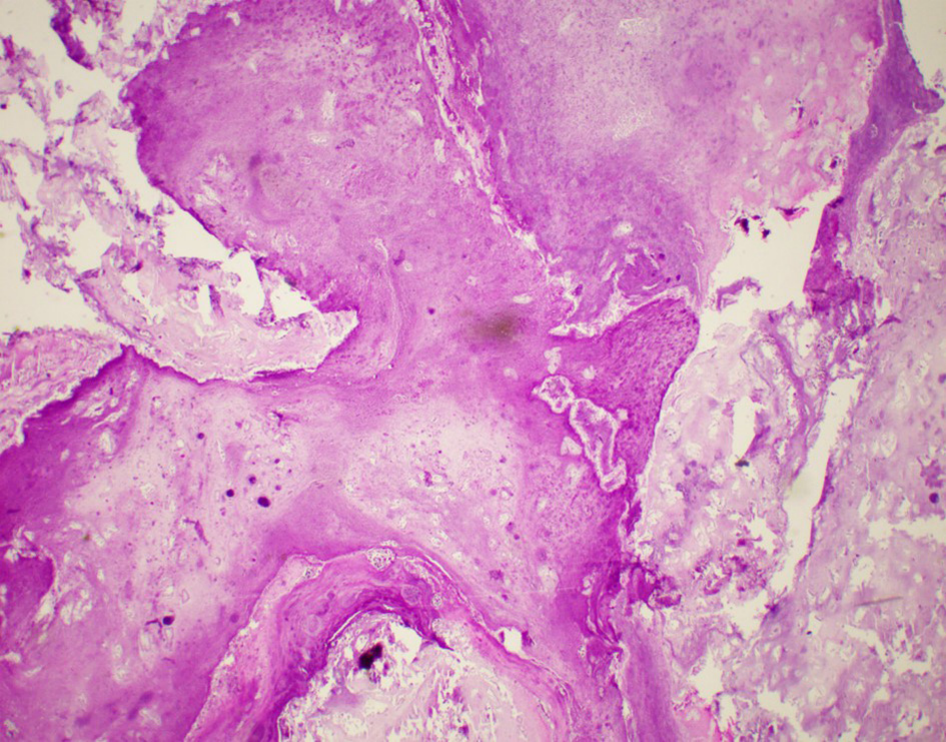
Hematoxylin and eosin stain at 20x magnification showing necrotic debris and some fibrinous exudates.

**Figure 7.**
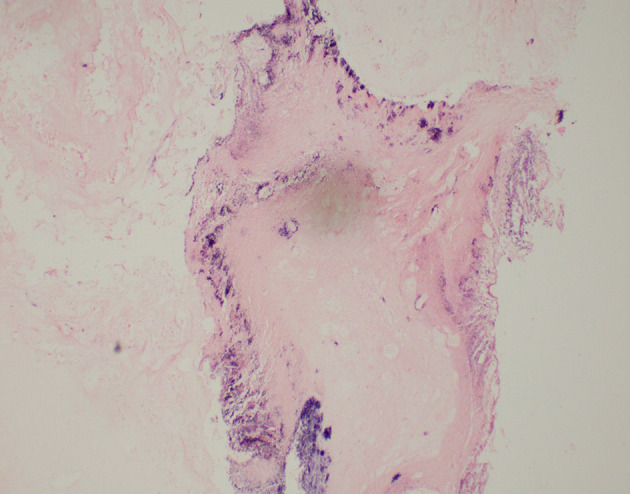
Gram stain at 40x magnification showing clumps of bacteria at periphery of lesion.

## Discussion

Bacterial ball has been reported by Kim et al in patients with chronic rhinosinusitis.^1^ Intraoperatively, the authors noted it is green and brown in colour, similar to fungal ball and difficult to differentiate from one another. Histologically, the matrix consists of thick acellular materials with bacterial colonies

Chrisholm and Hutch has first reported fungal balls within the urinary tract in 1961.^2^ Presence of air within the fungal ball gives rise to laminated appearance of fungus balls. There have been more than 20 cases been reported in the literature since then. Fungal urinary tract infections are most commonly caused by *Candida* species, but may also be caused by *Cryptococcus neoformans*, *Aspergillus* species and endemic mycoses.^3^

The most common predisposing factor for fungal urinary tract infection is diabetes mellitus.^3^ Other associated conditions include prolonged antibiotic therapy, use of corticosteroids or immunosuppressive agents, urinary stasis, and indwelling intravenous or urinary catheters.^4^ There are multiple mechanism which explains why diabetic contributing to increased risk of urinary tract infection. Immune system impairment and high tissue glucose levels in poorly controlled diabetic patients create a favourable environment for the growth and multiplication of microorganisms.^5,6^,^7^

Michigan postulated that in patient with renal candidiasis, pseudohyphae are seen growing into and within tubules.^5^ This causes marked inflammatory reaction and necrotizing papillitis. These pseudohyphae eventually forms obstructing fungal within the collecting system. In our case, we hypothesize bacterial ball forms from agglutination of necrotic tissue material, mucous and foreign debris, which is similar to fungal ball. The risk factor for formation of bacterial ball in our patient is likely multifactorial; diabetes mellitus, neurogenic bladder and long-term indwelling urinary catheter.

Fungal and bacterial balls may prove to be a diagnostic challenge on conventional imaging. Experience on CT to diagnose such lesions are limited in view of rarity of the disease. Takemura et al has reported a case of fungal ball mimicking urothelial carcinoma on MRI.^8^

Another possible differential diagnosis in our case is bacterial wall abscess. This condition is rare and has been associated with chronic urinary tract infection, history of permanent or intermittent catheterisation.^9,10^ Clinically, they present with intermittent fever and suprapubic pain. CT scan is almost diagnostic and reveals non-enhancing hypoattenuating content with enhancing peripheral rim.^9,10^ Cystoscopy often shows an elevated mass with inflamed overlying mucosa.^9,10^ Definitive treatment of bladder wall abscess requires drainage of the collection, which can be performed through transurethral route or percutaeneously under CT guidance.^9,10^

In our case, the bacterial ball was initially misdiagnosed as a gas containing bladder stone on CT scan. Conservative management will likely fail leading to recurrent haematuria and urinary tract infection. It is difficult to evacuate the bacteria ball by bedside manual bladder washout due to its consistency, size, and adherent nature. We propose direct visualisation, resection, and evacuation of the lesion. Microbiology laboratory findings and histopathology will help in confirming diagnosis of bacterial ball.

The further characterisation of MRI ﬁndings on fungal urinary tract infection is desired in order to assess the diagnostic power of this sophisticated device, as the diﬀerential diagnosis of fungus balls and neoplasms is sometimes challenging and can signiﬁcantly inﬂuence the clinical outcomes

## Conclusions

Bacterial ball in the bladder is a very rare entity. We report the first case in the English literature. Evacuation of the bacterial ball together with aggressive antibiotics therapy is effective and can lead to excellent outcomes.

## Learning points

1. Bacterial ball in bladder is a very rare cause of lower urinary tract infection
2. In rare cases, bacterial ball may develop in patients with neurogenic bladder and on long term indwelling urinary catheter placement
3. Bacterial ball may mimic a bladder stone on CT scan as it is hyperdense with Hounsfield unit of 420 units
4. Intraoperative findings of bacterial ball includes a soft mass with laminated appearance.
5. Preferred treatment will be evacuation of the bacterial ball, which may necessitate the use of a continuous flow resectoscope together with aggressive antibiotics therapy.
